# First experience with a supercharged pedicled jejunal interposition for esophageal replacement after caustic ingestion in a middle-income Latin American country

**DOI:** 10.1016/j.ijscr.2023.108293

**Published:** 2023-05-06

**Authors:** R. Alfaro-Pacheco, R. Brenes-Barrantes, J. Juantá-Castro, S. Rojas-Chaves, A. Echeverri-McCandless, P. Brenes-Barquero

**Affiliations:** a Servicio de Cirugía de Tórax, Hospital San Juan de Dios, Caja Costarricense de Seguro Social, Costa Rica; b Servicio de Cirugía Oncológica y Microcirugía, Hospital San Juan de Dios, Caja Costarricense de Seguro Social, Costa Rica; c Unidad de Investigación, Hospital San Juan de Dios, Caja Costarricense de Seguro Social, Costa Rica

**Keywords:** Caustic ingestion, Corrective surgery, Esophageal replacement, Pedicled jejunal bypass, Middle-income countries, Latin America

## Abstract

Caustic or corrosive substance ingestion that results in severe esophageal and gastric lacerations frequently requires surgical management. The most common sequelae after an upper gastrointestinal tract caustic injury include non-responding luminal strictures, which are subject to esophageal replacement. Late corrective surgery may include esophagectomy with gastric pull-up and jejunal or colonic interpositions. Although long-segment esophageal reconstruction with jejunum is technically feasible and has demonstrated good outcomes, the complexity of the surgery has precluded the widespread use of this procedure in low- and middle-income countries.

This document summarizes the most relevant aspects of caustic ingestion surgical management and describes the first Latin American experience in the reconstruction of an esophageal-gastric caustic injury using a pedicled jejunal interposition, as a viable and functional option in mid- and lower-income countries with well-established Thoracic Surgery departments and microsurgery access.

## Highlights

• Caustic or corrosive ingestion that results in severe esophageal and gastric lacerations, often require surgical management.
• Caustic injuries can cause small ulcerations to full-thickness lesions with necrosis and perforation of the digestive tube.
• Late correction surgery is reserved for severe cases were other alternatives fail or are not appropriate.
• Case report of the first experience in a middle-income country in Latin America.

## Graphical abstract

**Figure:**
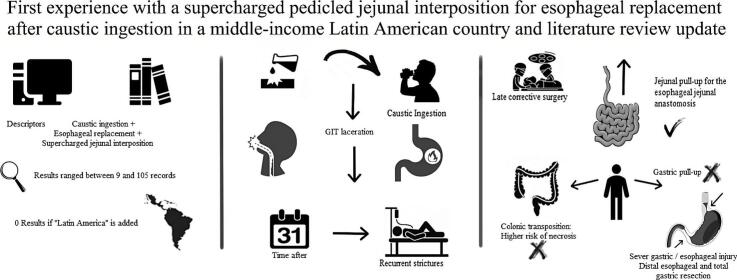

## 1 Introduction

Between 5000 and 18,000 cases of digestive tract lesions caused by ingestion of caustic or corrosive substances are reported yearly in the United States, from which around 80 % occur in young children, the remaining cases are mostly related to self-inflicted aggressions. Suicide attempt injuries of this nature are frequently life-threatening [1]. In developing countries, the incidence of severe caustic gastrointestinal lacerations is high and has been associated with social, economic, and educational factors. Despite efforts to regulate the situation and educate the population, this type of injury still constitutes an important and frequently unreported public health issue, especially in low- and middle-income countries [1], [2].

Suicide attempts are the most common reason for intended caustic material consumption and are usually seen in patients between 20 and 30 years of age with a previous history of psychiatric illnesses, behavioral or emotional afflictions, and substance use disorders [3]. Acids, alkalis, detergents, and peroxides account for the agents mostly associated with gastrointestinal tract injuries of this nature. Diluted aqueous hydrochloric acid solutions (muriatic acid) with concentrations ranging from 26,5 % to 38,0 % (*w*/w) have multiple domestic and industrial uses, are highly accessible to the population, and constitute a major alternative used for suicide attempts in developing countries [4]. Common concentrations of these solutions have pH values lower than 1 and cause extensive tissue damage. Occurring mostly in adults, Costa Rica's National Poison Control Center part of the Caja Costarricense de Seguro Social (CCSS), reports an increase in the number that hydrochloric acid intoxications during the last four years.

This work has been reported in line with the SCARE criteria. [5]

### 1.1 Acute gastrointestinal lesions

Immediate consequences of caustic injuries range from small ulcerations in the mucosa to full-thickness lesions with necrosis and perforation of the digestive tube [6]. A detailed explanation of the damage observed in the gastrointestinal tract after direct contact with caustic or corrosive elements, comprising from edema and hyperemia (class 1) to extensive necrosis (class 3B) has been described previously. Damage extent in this type of lesion varies according to numerous factors mainly related to the hazardous substance, which include: nature, physical form, the amount taken, concentration, time of contact, and the pre-ingestion condition of the tissue. Regarding the nature of the substance, acids in contrast to alkalis, produce an acute burning sensation along with immediate pain after contact with the oral mucosa; whereby ingested volumes of these substances tend to be low. As well, the acid's low viscosity makes a prompt upper gastrointestinal tract transit time [7].

Patients with clinical or radiological evidence of digestive tract perforation after the ingestion of the corrosive substance require expedited interventions, in which the extension of the procedures varies according to severity. Patients without features of perforation should be monitored because of the later development of necrosis, perforation, and massive bleeding. Immediate surgical management of caustic lacerations includes the resection of all injured organs, and regularly this proceeding has a negative impact on both survival and functional outcomes for the patient [8].

### 1.2 Late sequelae

Profound long-term pathological conditions resulting from caustic injuries include esophageal strictures or stenosis, abnormal constriction of the gastric antrum or pylorus (usually manifested as full stomach sensation, nausea, vomiting, and weight loss), gastrointestinal reflux, dysmotility, hemorrhage, fistula formation (tracheoesophageal, tracheobronchial, aortoenteric), pulmonary complications and esophageal or gastric carcinoma (cicatricial carcinoma) [6]. Esophageal carcinoma generally appears decades after the intake of the caustic or corrosive element and it has been reported in ranging frequencies between 2 and 30 %, which is 1000 to 3000 times higher when compared to the general population incidence rate. Gastric carcinoma as a result of caustic injury is rare [8].

Taking place around 8 weeks after the ingestion of the caustic substance and observed in up to 71 % of patients with grade 2B and more than 95 % of patients with grade 3 [9], esophageal strictures are the most common consequence of caustic injury. Endoscopic dilation, stents, and corrective surgery are the most used treatments for esophageal strictures. Recurrent endoscopic expansion procedures may cause esophageal perforation.

### 1.3 Main aspects of delayed reconstructive procedures

Late correction surgery is reserved for severe cases where other alternatives fail or are not appropriate. A stand-by period of 6 to 12 months post-corrosive ingestion is recommended before this type of intervention [10]. Considering the morbidity and mortality of the alternatives, there is no consensus if the best surgical approach for the management of corrosive strictures is the resection or bypass of the wounded segment. Although resection is associated with lower rates of esophageal carcinoma, this action is considered an independent negative predictor of survival [10]. The literature describes different routes and conduits used as part of the corrective surgery, each with its specific benefits and disadvantages.

The esophageal replacement may be accomplished by gastric advancement (pull-up), colonic transposition, or jejunal interposition. Usage of gastric conduits has become the first alternative in the restoration of the digestive tract after caustic injury, mainly because comparatively it is a less complicated procedure that only requires one anastomosis, which success rate is increased by the redundant blood supply of gastric tissue. Nevertheless, it is dependent on the availability of healthy stomach tissue [11], which may be difficult to determine in corrosive lesions [8], and long-term functional outcomes in gastric advancement surgery may be compromised with the development of complications such as stricture recurrence, reflux, and metaplasia over the anastomotic site [12].

Although esophagectomy with colon interposition is also a reconstructive surgery commonly done, mainly because the colon has abundant vascularity and a broad luminal space; it is a more challenging procedure. The presence of intrinsic pathologic conditions, the possible development of long-term functional problems like dysphagia, halitosis, reflux, bowel dumping, and diarrhea; the requirement of multiple anastomoses, and the fact that it can be influenced by issues such as colonic redundancy have led to the development of other alternatives [13]. Even though in the early 2000s clinical trials reported the superiority of left to right colonic interposition, nowadays the portion of the colon (left or right) used for esophageal reconstruction will depend on the length of the graft required, the intrinsic colonic vascular anatomy of the section under consideration and the surgeon's choice and experience. It has also been reported that colonic interposition, in comparison with gastric or jejunal reconstructions, may present slightly higher rates of necrosis and other complications [13], [14].

As gastric pull-ups, colonic transpositions commonly use the retrosternal route although the substernal and posterior mediastinum positions are also frequent [15]. While both reconstructive procedures have shown good outcomes with no significant mortality difference, colon interpositions report an increment in surgical times, blood loss, anastomotic leakages, and sepsis.

### 1.4 Jejunal interposition for esophageal replacement

The similarities in the luminal diameter (that reduces the possibility of organ compression), peristaltic activity (that may be favorable for deglutition), and structure between the esophagus and the jejunum, as well as the relative absence of intrinsic disease, jejunum abundancy [15] and the fact that tissue preparation does not require any formal processes, makes this section of the small intestine compatible for esophageal reestablishment. Although it is also considered a technically challenging and demanding procedure that requires accurate planning and a multidisciplinary approach [15], aspects that may constitute a challenge in low- and middle-income countries.

Pedicled jejunum has been used for esophageal reconstruction, although the replacement of the upper sections of the digestive tract involves the mobility and straightening of the jejunal tissue through a simple dissection of the mesentery and circulatory restoration achieved by anastomosing tissue veins and arteries with vessels from the upper thorax or neck through microvascular techniques (supercharge) [15]. These procedures allow elongation, a tension-free transposition, reduce flap necrosis, and create vigorous perfusion at the anastomotic site that reduces leaks [16].

The type of replacement determines if a supercharged pedicled jejunal flap (augmented with cervical or thoracic vascular micro anastomosis) or a free jejunal transfer graft is needed, reconstruction of long segments usually requires microvascular augmented pedicled flaps, meanwhile, free transfers are used for reconstruction of small esophageal or pharyngeal defects (usually 15 cm or shorter) [16]. Over time, jejunal pull-up surgeries report an anastomotic leakage rate close to 20 %, 8 % of graft loss and around 90 % of patients return to oral nutrition before a 6-month period [17].

As mentioned before, worldwide gastric pull-ups and colonic interpositions account for the most frequent esophageal replacement surgeries, nevertheless, pedicled jejunal interpositions with microvascular augmentation constitute well-known and regularly used interventions in European, Asian, and North American developed countries. The need for advanced technology, major surgical skills [18], [19], and training, as well as the economic cost of long jejunal interpositions, represent mid- and lower-income countries highly restrict the implementation and experience of this procedure in developing nations. After reviewing published literature and to the best of our knowledge, this paper incorporates the first description of a supercharged pedicled jejunal interposition for esophageal replacement after caustic ingestion in a low- or middle-income Latin American country.

## 2 Case report

### 2.1 Medical history and sociodemographic characteristics

A young male patient from a reduced socioeconomic indicators urban district, who has no reported chronic medical illness, and no evidence of substance use disorder, the patient has a personal and familial history of mental illnesses, recurrent suicide attempts, and self-inflicting injuries. Despite intermittent psychiatric consultations, he was not undergoing any psychiatric treatment at the time of muriatic acid consumption.

### 2.2 Clinical manifestations and management

Described male experiencing oropharyngeal, retrosternal, and epigastric pain was admitted to a Caja Costarricense del Seguro Social referral hospital's Emergency Department after the ingestion of muriatic acid as part of a suicide attempt. At presentation, the patient was conscious and had shortness of breath, dysphagia, and salivation. After airway stabilization and decontamination, potential injuries of the upper respiratory tract were evaluated and no need for intubation was determined. Image evaluation revealed no imminent signs of perforation or bleeding, and dilution and neutralization therapies were administered. Because of the extent of the lesions, the overall condition of the patient, the medical history, and the risk of perforation and massive bleeding, the patient was transferred to the Intensive Care Unit (ICU). Additionally, systemic steroids to inhibit inflammation and reduce potential stricture formation and antibiotics to counter possible esophagus infection were used. Once discharged from the ICU, psychiatric and gastrointestinal evaluations, along with social services assessments were performed. The patient was diagnosed with depressive disorder and borderline personality traits, valproic acid and lamotrigine were prescribed. After an adequate follow-up period in which symptoms and lesions did not show progression and the tolerability of oral fluids and food was confirmed, the patient received the hospital discharge.

Three weeks after the ingestion of the caustic substance, the patient returned to the hospital referring to full stomach sensation, nausea, and vomiting. Endoscopy revealed esophageal strictures and an immediate dilation procedure was accomplished.

**Fig. 1:**
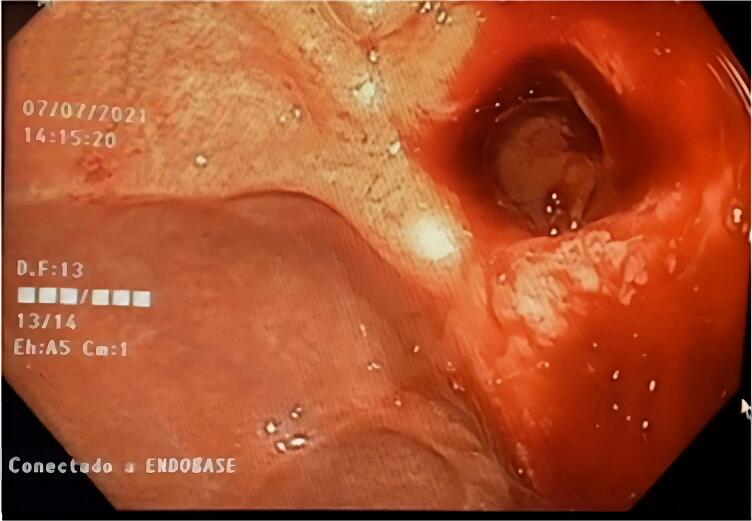
Upper gastrointestinal endoscopy showing a severe inflammatory process.

New attention at the Emergency Department two months after the initial discharge, this time with epigastric and chest pain, esophagitis, vomiting, dysphagia, and the inability to tolerate fluids is also reported. The Gastroenterology Department reassesses the patient and endoscopic results identified a severe inflammatory process, including edema, diffuse congestion of the esophagus (Fig. 1), and linear erosions covered with fibrin throughout the tissue. No ulcers or definitive areas of necrosis were observed, and findings are consistent with a Zargar classification type 2A.

**Fig. 2:**
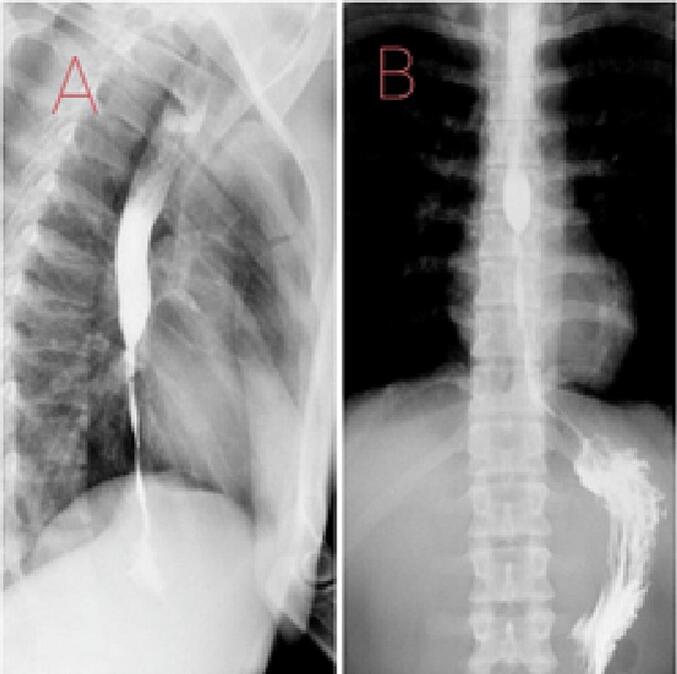
Esophagogram showing cicatricial scarring of the distal esophagus with severe stenosis. A. lateral view. B. Antero-posterior view.

Filiform stenosis that precluded endoscope passage was also described and confirmed through an esophagogram. Esophagogram results documented abrupt esophageal stenosis at T8, which ranged from 25 mm in diameter in the most proximal segment of the constriction to 5 mm in the thinnest section. The luminal stricture allowed transit and the contrast medium formed an irregular filiform path of approximately 95 mm in length into the esophageal-gastric junction (Figs. 2 A and B).

Further endoscopic results included the loss of gastric distensibility, alterations to the gastric mucosa as a result of numerous scars, and pyloric stenosis that hindered the endoscope's passage. After dilatation of this section, a duodenal stent was placed and removed before the corrective surgical intervention.

### 2.3 Esophageal replacement surgery

**Fig. 3:**
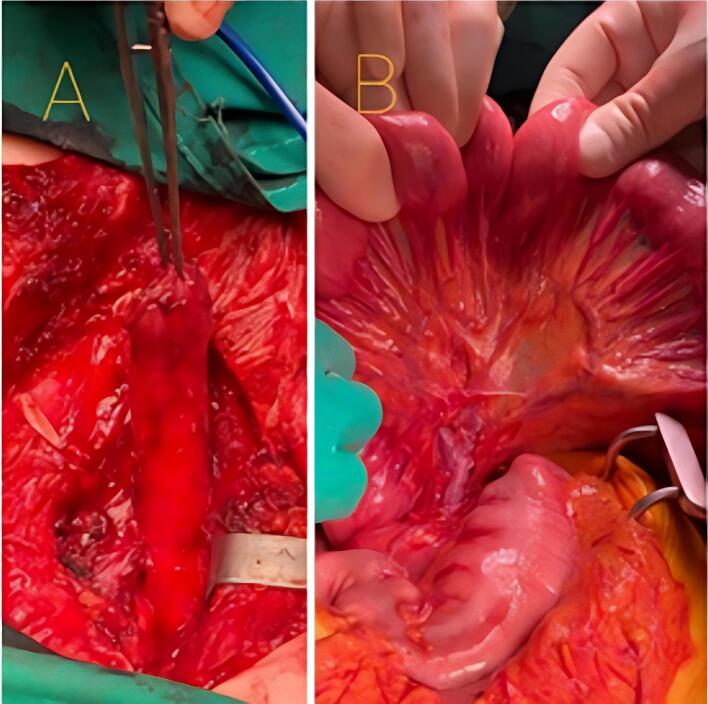
Esophageal replacement surgery tissue preparation for supercharged pedicled jejunal interposition A. Esophagus remnant. B. Proximal jejunum vasculature assessment.

Over a right posterolateral thoracotomy approach, the patient's esophagus was dissected, and the azygos vein was ligated and cut. The proximal remaining section of the esophagus was fastened approximately 40 mm away from the cricoid cartilage (where healthy tissue was evident) and left for the succeeding manual anastomosis with the jejunal tissue (Fig. 3A). Subsequently, a total gastric resection and simultaneous cholecystectomy through midline incision laparotomy were conducted. In addition, transillumination of the mesentery to evaluate the arcades of the proximal jejunal tissue, which would later be used for the pull-up, was performed (Fig. 3 B).

**Fig. 4:**
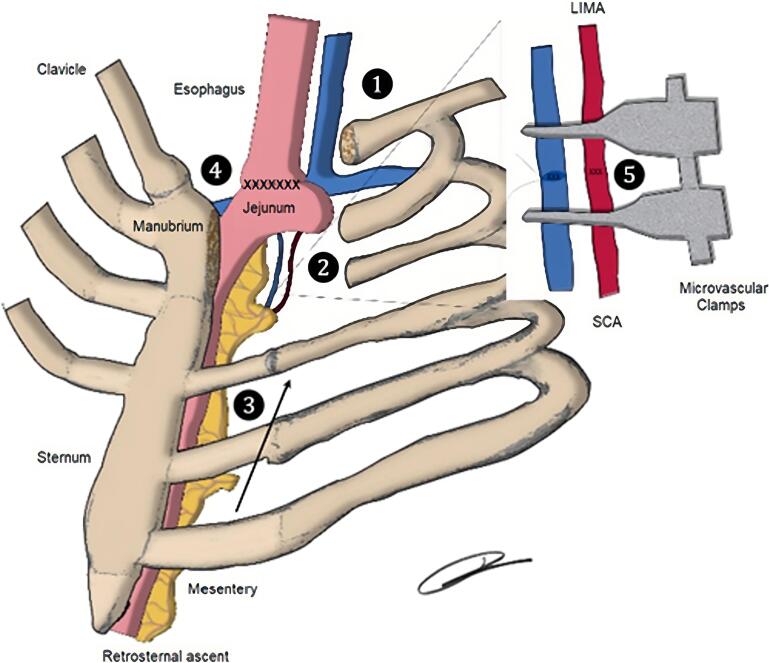
Esophageal replacement thoracic cage anatomic relationships. 1) Partial resection of the clavicle's medial third 2) Costal cartilages and manubrium dissection. 3) Jejunal pull-up 4) Esophageal jejunal surgical connection 5) Microanastomosis between LIMA and the second branch of the superior mesenteric artery (SMA).

A cervical incision over the sternocleidomastoid muscle straight to the thoracic midline was executed, upon the reach of the plane under consideration, a slight rotation was made, and the incision extended for around 80 mm below the suprasternal notch. Elevation of the musculocutaneous flap product of the previous dissection exposed the medial third of the clavicle, the manubrium, and the sternal body. Resection of the clavicle's medial third, the first two costal cartilages, and the manubrium's left half (Fig. 4), in addition to the dissection of the retrosternal space, exposed the esophageal stump and created a tension-free space for the succeeding esophageal jejunal surgical connection and necessary microvascular anastomoses.

**Fig. 5:**
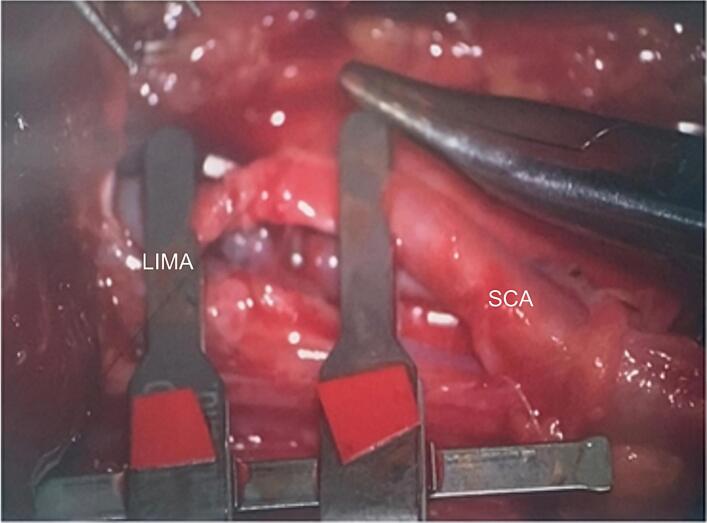
Microanastomosis of the vascular pedicle of the second jejunal branch with internal mammary vessels.

The proximal jejunum was released near the second arch and using a plastic laparoscopic bag, tissue ascent was performed transmesocolic and retrosternaly. Microsurgical anastomoses between the left internal mammary artery (LIMA) and the second jejunal branch of the superior mesenteric artery (SMA), as well amongst the left internal mammary vein (LIMV) and the jejunal vein were performed using a Zeiss OPMI Pentero 800 surgical microscope. Other microsurgical instruments used included microvascular clamps and sutures (Fig. 4, Fig. 5).

Using a continuous monofilament suture an end-to-side anastomosis between the esophageal stump and the pulled-up jejunal tissue was performed, and the cervical area was closed leaving a Jackson-Pratt drain. At the abdomen, anastomosis was performed a for Roux - en - Y reconstruction, between the jejunal loop used for ascent and the remaining jejunal loop (duodenum distal section). Prior to the closure of the abdominal cavity, a jejunostomy was performed and a Jackson-Pratt drain was left in place.

No immediate postoperative complications were reported, and trans-operative bleeding was minimal. The surgery lasted around 12 h and involved more than 20 different specialists.

### 2.4 Postoperative follow up

Postoperative follow-up was made 1 month after surgery, the patient was eating a normal diet, and the feeding jejunostomy tube was removed with no complications.

## 3 Discussion

Commonly caustic ingestion sequelae require corrective surgery to maintain the digestive tract continuity and function. Esophageal reconstruction is one of the most challenging procedures that a thoracic surgeon can face [13], [18]. In this situation, the main aspects that must be considered when selecting the appropriate interventional approach include the nature and extent of the lesions, failure of previous treatment alternatives, absence of other management options, the burden of the disease, patient's intrinsic conditions, lifestyle, and cancer risk as a result of caustic exposure. We cannot refrain from mentioning the variable impact that the selected surgical technique may have on the quality of life of the patient, in addition to the possibility of recurrent complications, necrosis, and malignancy risk [13].

Another aspect that must be taken into consideration is the capability of the health center to perform the selected surgery [20]. In the hospital setting the achievement of any given procedure is influenced and determined by a series of technical and administrative conditions. In our case, within a public social security health system in a middle-income country that covers practically the entire population, frequent financial limitations and prioritization of resources commonly restrict involvement and experience development in complex, high-priced, advanced procedures. Although improvements in surgical techniques and perioperative management, the existence of highly trained personnel and the accessibility to microsurgical technology enabled the initial development of advanced procedures at the biggest national adult reference health center in Costa Rica.

In the pertaining case, considering the severity of the lesions, the patient's age, the recurrent requirement of multiple endoscopic interventions, the possibility of perforations secondary to expansion procedures, the previous failure of other alternatives, and the substantial risk of developing malignant lesions in the future; surgical management with enteral pathway reconstruction was decided. Since gastric tissue laceration was previously described, given the higher rate of necrosis and other complications with colonic interposition, and considering the hospital's surgical advancement, for the first time in the region a supercharged pedicled jejunum interposition was conceived, proposed, and successfully implemented.

The description of this case also enables the comparison of coincidences and differences with other centers that have greater experience in the employed technique and larger financial resources, nevertheless, the biggest relevance of this report lies in the fact that it can be used as an example in other low-and middle- income countries for the possible implementation of a significant alternative in patients requiring esophageal replacement.

## 4 Conclusion

Although long-segment esophageal reconstruction using a supercharged pedicled jejunal interposition is a technically feasible procedure that has demonstrated good outcomes in patients with esophageal-gastric caustic damage, the complexity of the intervention and the requirement of microsurgical technology has precluded its extensive use, mostly in mid- and lower-income countries. This report demonstrates that esophageal replacement using a jejunal conduit can be achieved with good results in countries where healthcare resources are limited if a multidisciplinary approach led by Thoracic Surgery departments with microsurgery access is implemented. The context previously explained in this paper is of great importance because, in the nearby future, developing countries could offer patients a new surgical alternative so further research could help in overcoming this challenges.

## Funding sources

This research did not receive any specific grant from funding agencies in the public, commercial, or not-for-profit sectors.

## Informed consent

Written informed consent was obtained from the patient for the publication of this case report and accompanying images. A copy of the written consent is available for review by the Editor-in-Chief of this journal on request.

## Declaration of competing interest

The authors have no conflict of interests to declare.
